# Coding-Complete Genome Sequences of Three SARS-CoV-2 Strains from Bangladesh

**DOI:** 10.1128/MRA.00764-20

**Published:** 2020-09-24

**Authors:** Shahina Akter, Tanjina Akhtar Banu, Barna Goswami, Eshrar Osman, Mohammad Samir Uzzaman, M. Ahashan Habib, Iffat Jahan, Abu Sayeed Mohammad Mahmud, M. Murshed Hasan Sarker, M. Saddam Hossain, A. K. Mohammad Shamsuzzaman, Tasnim Nafisa, M. Maruf Ahmed Molla, Mahmuda Yeasmin, Asish Kumar Ghosh, Sheikh M. Selim Al Din, Utpal Chandra Ray, Salek Ahmed Sajib, Maqsud Hossain, M. Salim Khan

**Affiliations:** a Bangladesh Council of Scientific and Industrial Research, Dhaka, Bangladesh; b SciTech Consulting and Solutions, Dhaka, Bangladesh; c National Institute of Laboratory Medicine and Referral Center, Dhaka, Bangladesh; d Invent Technologies, Ltd., Dhaka, Bangladesh; e NSU Genome Research Institute (NGRI), Department of Biochemistry and Microbiology, North South University, Dhaka, Bangladesh; Queens College

## Abstract

We report the sequencing of three severe acute respiratory syndrome coronavirus 2 (SARS-CoV-2) genomes from Bangladesh. We have identified a unique mutation (NSP2_V480I) in one of the sequenced genomes (isolate hCoV-19/Bangladesh/BCSIR-NILMRC-006/2020) compared to the sequences available in the Global Initiative on Sharing All Influenza Data (GISAID) database. The data from this analysis will contribute to advancing our understanding of the epidemiology of SARS-CoV-2 in Bangladesh as well as worldwide at the molecular level and will identify potential new targets for interventions.

## ANNOUNCEMENT

Severe acute respiratory syndrome coronavirus 2 (SARS-CoV-2) belongs to the genus *Betacoronavirus*, family *Coronaviridae*, and is the causative agent of the ongoing coronavirus disease 2019 (COVID-19) pandemic (https://coronavirus.jhu.edu/map.html). SARS-CoV-2 was first reported in Bangladesh in March 2020, and since then the country has experienced a steady rise in infections, resulting in ∼115,786 cases and 1,502 deaths as of 22 June 2020 (https://corona.gov.bd/?gclid). Here, we report the complete sequences of three SARS-CoV-2 isolates from patients who tested positive using quantitative PCR (qPCR) in the National Institute of Laboratory Medicine and Referral Center (NILMRC). qPCR was performed using a Sansure Biotech novel coronavirus (2019-nCoV) nucleic acid diagnostic kit according to the manufacturer’s instructions. The threshold cycle (*C_T_*) values of the N gene of the three positive samples were 24.3, 22.6, and 23.4, and the *C_T_* values of the open reading frame (ORF) genes were 24.9, 27.5, and 22.6, respectively.

The study was approved by the ethics committee of NILMRC (Bangladesh). The samples were taken with the consent of all patients, and they provided informed written consent consistent with the experiment. To understand the molecular epidemiology of SARS-CoV-2 viruses in Bangladesh, we carried out whole-genome sequencing of three isolates (hCoV-19/Bangladesh/BCSIR_NILMRC_006/2020 [BCSIR_NILMRC_006], hCoV-19/Bangladesh/BCSIR_NILMRC_007/2020 [BCSIR_NILMRC_007], and hCoV-19/Bangladesh/BCSIR_NILMRC_008/2020 [BCSIR_NILMRC_008]) collected directly from nasopharyngeal swabs from three patients in Bangladesh. Viral RNA was extracted using a PureLink viral RNA/DNA minikit (catalog no. 12280050; Thermo Fisher Scientific, USA). The cDNA of all three samples was used to prepare paired-end libraries with the Nextera DNA Flex library preparation kit according to the manufacturer’s instructions (Illumina, Inc., San Diego, CA). The extracted viral RNA was converted into cDNA using the GoScript reverse transcription system protocol according to the manufacturer’s instruction. The library pool of three samples was sequenced using a NextSeq high-output kit with an Illumina NextSeq 550 instrument in paired-end format (read length, 151 bp).

**FIG 1:**
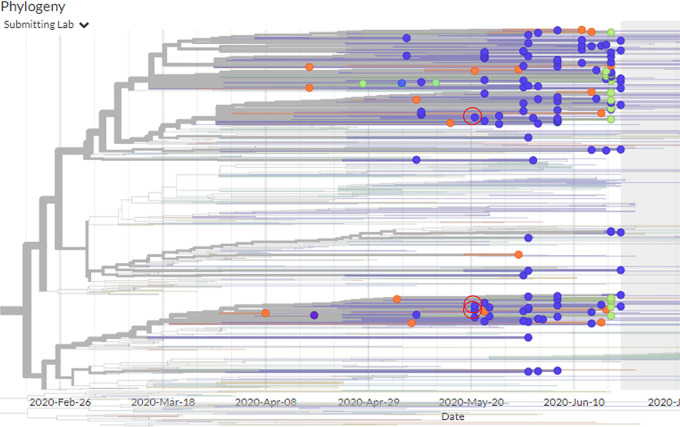
Phylogenetic tree of SARS-CoV-2 using isolates BCSIR_NILMRC_006, BCSIR_NILMRC_007, and BCSIR_NILMRC_008 in Bangladesh in May and June 2020. The *x* axis represents the number of mutations from the Wuhan strain (GenBank accession no. MT539159, MT539158, and MT539160). The red circles represent the respective positions of the three isolates used in this study along with other Bangladeshi genomes available in GISAID. The figure was condensed using Nextstrain (3) (https://nextstrain.org/ncov/global?dmax=2020-06-10&f_country=Bangladesh).

The library generated 73,164,498, 109,775,424, and 70,072,652 reads, of which 72,000,769, 106,842,322, and 68,5498,53 unique reads, respectively, were found after excluding duplicate marked reads using BaseSpace DRAGEN RNA Pathogen Detection software version 3.5.1 (1). Sequence trimming and quality control were done using the BaseSpace DRAGEN app. Quality control examination of the sequencing reads revealed that >99% of the sequencing data yielded a Phred score of 30 or above. We did not identify any overrepresented adapter sequences in the sequencing libraries. After generating a FASTA file from the FASTQ files using the DRAGEN software, it was found that the complete genome sequences of the Bangladeshi SARS-CoV-2 strains (BCSIR_NILMRC_006, BCSIR_NILMRC_007, and BCSIR_NILMRC_008) have linear RNAs of 29,892 bp, 29,823 bp, and 29,758 bp, respectively, with an average GC content of 39%. Data for the three samples were uploaded to the Global Initiative on Sharing All Influenza Data (GISAID) database on 30 May 2020 (2). Phylogenetic analysis of these three virus genomes grouped them in SARS-CoV-2 clade 20B (Fig. 1).

This phylogeny shows the evolutionary relationships of human coronavirus 2019 (hCoV-19; or SARS-CoV-2) viruses from the ongoing COVID-19 pandemic. The results show an initial emergence in Bangladesh in May and June 2020, followed by sustained human-to-human transmission, leading to the sampled infections.

Compared with hCoV-19/Wuhan/WIV04/2019, for strain BCSIR-NILMRC-006, we found eight mutations, including NSP2_G339S, N_R203K, N_G204R, NSP3_Q172R, Spike_D614G, NSP2_I120F, NSP12_P323L, and NSP2_V480I. Six mutations were found in BCSIR-NILMRC-007, Spike_D614G, N_R203K, N_G204R, NSP12_K59N, NSP2_I120F, and NSP12_P323L. In BCSIR-NILMRC-008, the genome mutations Spike_D614G, N_R203K, N_G204R, NSP2_I120F, NSP12_P323L, and NSP3_P822S were observed (Table 1). A unique mutation (NSP2_V480I) was observed in the BCSIR-NILMRC-006 genome sequence compared to the genome sequences available in the GISAID CoVsurver (GISAID Initiative_CoVsurver_files).

**TABLE 1:** Mutations present in the three SARS-CoV-2 strains isolated in Bangladesh (GISAID Initiative_CoVsurver_files)^*a*^

Isolate name	Length (nt)^*b*^	Length (aa)^*c*^	No. of mutations	Unique mutation	Existing mutations	Clade
hCoV-19/Bangladesh/BCSIR_NILMRC_006/2020	29,892	9,710	8	NSP2_V480I	NSP2_G339S, NSP2_I120F, NSP3_Q172R, NSP12_P323L, Spike_D614G, N_G204R, N_R203K	GR
hCoV-19/Bangladesh/BCSIR_NILMRC_007/2020	29,823	9,710	6		NSP2_I120F, NSP12_P323L, NSP12_K59N, Spike_D614G, N_G204R, N_R203K	GR
hCoV-19/Bangladesh/BCSIR_NILMRC_008/2020	29,758	9,710	6		NSP2_I120F, NSP3_P822S, NSP12_P323L, Spike_D614G, N_G204R, N_R203K	GR

a Based on the reference sequence hCoV-19/Wuhan/WIV04/2019.

b nt, nucleotides.

c aa, amino acids.

### Data availability.

The sequences of these SARS-CoV-2 genomes from Bangladesh were submitted to the GISAID database (accession no. EPI_ISL_455458, EPI_ISL_455420, and EPI_ISL_455459) and GenBank (accession no. MT539159, MT539158, and MT539160). The raw reads have been deposited in the BaseSpace cloud and were also submitted to the NCBI SRA under accession no. PRJNA658211, PRJNA657985, and PRJNA657938 for strains BCSIR_NILMRC_008, BCSIR_NILMRC_007, and BCSIR_NILMRC_006, respectively.
